# Diabetes associated with cervical carcinoma among high-risk HPV-infected patients with cytologically diagnosed high grade squamous intraepithelial lesion

**DOI:** 10.3389/fendo.2022.993785

**Published:** 2022-10-27

**Authors:** Chaoyan Yue, Chunyi Zhang, Chunmei Ying, Hua Jiang

**Affiliations:** Obstetrics and Gynecology Hospital of Fudan University, Shanghai, China

**Keywords:** cervical cancer, hgsil, hpv, HbA1c, diabetes

## Abstract

**Background:**

Diabetes causes metabolic disorders and immune changes that may be potential triggers of cervical cancer. Therefore, diabetes is not a “bystander” to cervical cancer. However, the conclusion that diabetes promotes cervical cancer lacks clinical epidemiological evidence, and the reported potential association between diabetes and cervical cancer is controversial.

**Methods:**

We conducted an explorative cross-sectional study of 791 women with cytological HGSIL and HR-HPV, who attended the cervical clinic of the largest academic women’s hospital in China from May 2019 to March 2022. After cervical screening, patients who were requiring colposcopy were tested for HbA1c. HbA1c level of 6.5% or higher defines diabetes and HbA1c level of 5.7%-6.4% was defined as prediabetes. The relationship between diabetes and cervical cancer was observed by a dose-response graph. Subgroup analysis and multivariate logistic regression analysis were conducted to estimate the associations between diabetes and cervical cancer.

**Results:**

Among HGSIL patients with high-risk HPV infection, compared with women with HbA1c <5.7%, the odds ratio for women with prediabetes was 1.72 (95% CI: 0.87-3.41) and the odds ratio for women with diabetes was 3.29 (95% CI: 1.10-9.80) for cervical cancer. Sensitivity analysis showed that diabetes was significantly associated with cervical cancer in different age groups and different HPV variant. E-value analysis showed robustness to unmeasured confounding.

**Conclusions:**

In patients with HR-HPV combined with HGSIL, diabetes and prediabetes are associated with cervical cancer.

## Introduction

The global prevalence of diabetes has risen dramatically, affecting more than 8% of adults worldwide (1). Data from China for 2018 show that the prevalence of diabetes has increased to 12.4%, with the prevalence of antecedent diabetes reaching 38.1%, diabetes and prediabetes 50.5%, and a weighted percentage of women 49.5% (2). Hemoglobin A1c (HbA1c) is the current gold standard for monitoring glycemic control and is now recommended for diagnosing diabetes and identifying individuals at risk of developing diabetes (3). HbA1c reflects the average blood glucose level over the past 2-3 months. Compared to fasting blood glucose (FPG), HbA1c is easy to measure (no fasting required), has minimal daily variability, greater pre-analytical stability, and fewer daily interruptions during stress, diet, or illness. Does not require rapid or timed sampling; measurements are standardized and correlate more closely with chronic complications (4).

According to the World Health Organization, cervical cancer ranks as the second most common cancer among women worldwide, with an annual incidence of 5.28 million and a mortality rate of 2.66 million. Approximately 87% of these deaths occur in women in developing countries. Human papillomaviruses (HPV16 and HPV18) are strongly associated with the development of cervical cancer in women (5). Diabetes causes metabolic disorders and immune changes that may be potential triggers of cervical cancer (6, 7). Therefore, diabetes is not a “bystander” to cervical cancer. However, the conclusion that diabetes promotes cervical cancer lacks clinical epidemiological evidence, and the reported potential association between diabetes and cervical cancer is controversial (8–18).

To elucidate the association of cervical cancer in women with diabetes or prediabetes, our cross-sectional study used HbA1c as a diagnostic criterion for diabetes, and explored the association of diabetes with cervical cancer in patients with high-risk HPV infection and cytologically diagnosed high grade squamous intraepithelial lesion (HGSIL) relation.

## Methods

Our study was an explorative cross-sectional study with a total of 791 women between May 2019 and March 2022 at the Obstetrics & Gynecology Hospital of Fudan University (Shanghai, China), which was China’s largest academic woman’s hospital. According to the American Diabetes Association, an HbA1c level of 6.5% or higher defines diabetes. An HbA1c level of 5.7%-6.4% was defined as prediabetes. History of adverse pregnancy includes miscarriage, intrauterine embryo arrest, malformation and stillbirth. Patients at high-risk for cervical cancer defined as women who had cytological HGSIL and tested positive for HR-HPV. Inclusion criteria: HPV-DNA examination results of HPV16, HPV 18 or other high-risk types(HPV31, HPV33, HPV35, HPV39, HPV45, HPV51, HPV52, HPV56, HPV58, HPV59, HPV66, HPV68). HGSIL was diagnosed by Pap smear. Clinical data and results were obtained from Hospital Information System (HIS) and Laboratory Information Management System (LIS). The study was approved by Ethics Committee of the Obstetrics & Gynecology Hospital of Fudan University(2022-78). The data are anonymous, and the requirement for informed consent was therefore waived.

### Variables and measurements

After cervical screening, patients who were requiring colposcopy were tested for HbA1c. HbA1c results were used as exposure factors in this study. HbA1c was analyzed using the Hemoglobin Testing System (VARIANT II, Bio-Rad). Confounding factors included age, BMI, history of adverse pregnancy, vaginitis, and HPV type.

HPV testing was performed in the pathology department with 1 of 2 hrHPV testing methods during the study period: the Cobas 4800 system (Roche Diagnostics), or BioPerfect (Bioperfectus Technology).

### Outcomes and measurements

The outcome was cervical cancer, and the diagnosis was based on histological pathological findings. Cases with histopathologic results that were diagnosed in our department after a Pap smear result of HGSIL were selected for analysis. Histopathological tissue acquisition procedures included cervical biopsy, cervical canal scraping, diagnostic hysterectomy by loop electrosurgical excision procedure or cold knife conization, and hysterectomy. The first histologic follow-up for most patients was a cervical biopsy. More severe lesions were recorded if more than 1 follow-up result was found.

### Statistical analysis

Data are expressed as mean (SD) for continuous variables and as percentage (%) for dichotomous variables. A smoothed curve fit plot of HbA1c was created to examine the shape of the relationship between HbA1c and cervical cancer. Logistic regression models were used to examine the effect of diabetes and other variables on cervical carcinogenesis. We performed tests for linear trend by entering the median value of each category of HbA1c as a continuous variable in the models. These models were adjusted for age, history of abnormal pregnancy, vaginitis, and HPV type. The robustness of these findings was assessed in multiple sensitivity analyses. First, subgroup analyses were performed according to age and HPV type as stratifying factors, and interaction tests assessed whether the association between diabetes and cervical cancer was consistent between subgroups. Second, we explored the possibility of unmeasured confounders between diabetes and cervical cancer by calculating E values (19). E values quantify the magnitude of unmeasured confounders that may negate the observed association between diabetes and cervical cancer. A P value <0.05 was considered statistically significant. All reported P values are two sided. Statistical analyses were performed using R version 3.5.1 (R Foundation for Statistical Computing) and the software IBM SPSS (version 21.0. IBM; Armonk, NY)

## Results

**Figure 1:**
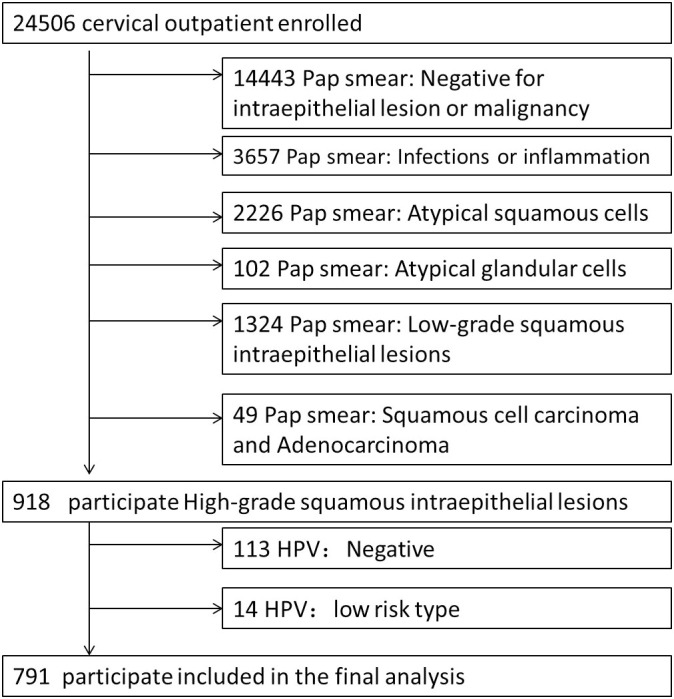
Flowchart of Participants.

Our study included 791 high-risk patients with cervical cancer (**Figure 1**). The average age of 43.95 ± 11.36. The mean glycated hemoglobin was 5.49 ± 0.56%, and the number of cervical cancer cases diagnosed by histopathology was 49 (6.19%). **Table 1** describes the baseline characteristics of the subjects, as well as some clinical characteristics that may be related to the occurrence of cervical cancer, such as age, HPV infection type, adverse maternal history and vaginitis.

**Table 1:** Baseline characteristics of the study participants.

	HbA1c<5.7	5.7≤HbA1c<6.5	HbA1c≥6.5	*p*-value
No. of participants	564 (71.30%)	196 (24.78%)	31 (3.92%)	
age	41.04 ± 10.08	50.23 ± 10.73	57.00 ± 12.12	<0.001
HbA1c	5.24 ± 0.28	5.91 ± 0.19	7.35 ± 0.81	<0.001
cervical carcinoma				<0.001
0	540 (95.74%)	177 (90.31%)	25 (80.65%)	
1	24 (4.26%)	19 (9.69%)	6 (19.35%)	
HPV type				0.591
Other high-risk types	276 (48.94%)	99 (50.51%)	18 (58.06%)	
16/18	288 (51.06%)	97 (49.49%)	13 (41.94%)	
History of adverse pregnancy				0.301
0	344 (60.99%)	117 (59.69%)	23 (74.19%)	
1	220 (39.01%)	79 (40.31%)	8 (25.81%)	
Vaginitis				0.52
0	537 (95.21%)	188 (95.92%)	29 (93.55%)	
1	27 (4.79%)	8 (4.08%)	2 (6.45%)	

0=NO, 1=Yes.

**Figure 2:**
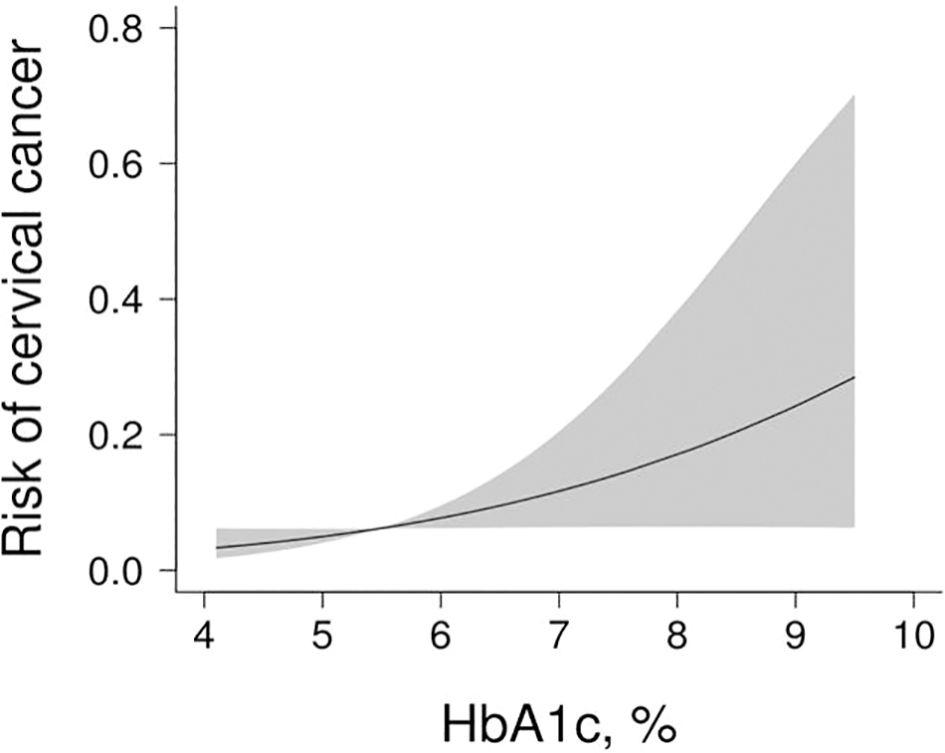
The association between HbA1c and cervical cancer. **Figure 2** adjusted for age, adverse maternal history, vaginitis, and type of HPV infection. The red line represents the fitted curve of HbA1c and cervical cancer, and the blue line represents the 95% confidence interval of the curve.

The dose-response relationship between HbA1c and cervical cancer was shown by smoothed splines (**Figure 2**). After adjustment for confounders, the odds ratio for cervical cancer in women with prediabetes was 1.72 (95% CI: 0.87-3.41) compared with women with HbA1c <5.7%, *p* value for trend=0.0001, and the odds ratio for cervical cancer in women with diabetes was 3.29 (95%CI: 1.10-9.80), *p* value for trend=0.0247 (**Table 2**).

**Table 2:** Individual effect of HbA1c on cervical carcinoma.

**Exposure**	**Incidence, n (%)**	**Non-adjusted**		**Adjust model I**	
		**OR (95% CI)**	***p* value**	**OR (95% CI)**	***p* value**
Continuous HbA1c	49 (6.19%)	1.98 (1.36, 2.88)	0.0003	1.58 (1.02, 2.44)	0.0408
Clinical cutoffs					
HbA1c <5.7	24 (4.26%)	Reference		Reference	
5.7≤HbA1c<6.5	19 (9.69%)	2.42 (1.29, 4.51)	0.0057	1.72 (0.87, 3.41)	0.1196
HbA1c≥6.5	6 (19.35%)	5.40 (2.03, 14.39)	0.0007	3.29 (1.10, 9.80)	0.0328
p value for trend			0.0001		0.0247

Ajusted for age, history of adverse pregnancy, vaginitis, HPV type.

**Figure 3:**
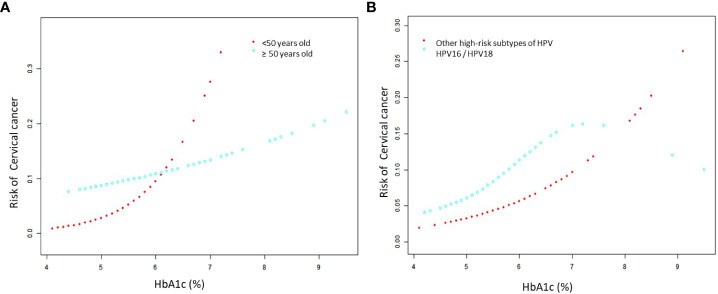
The association for subgroup analysis between HbA1c and cervical cancer. **(A)** Smooth fitting curve stratified by age, adjusted for adverse maternal history, vaginitis, and type of HPV infection. **(B)** Smooth fitting curve stratified by type of HPV infection, adjusted for age, adverse maternal history and vaginitis.The red line represents the fitted curve of HbA1c and cervical cancer, and the blue line represents the 95% confidence interval of the curve.

Sensitivity analyses by age and HPV type found that the effect of HbA1c on cervical cancer was consistent between subgroups. There was no interaction between the different subgroups (age p=0.0748 for the interaction; HPV type: p=0.8964 for the interaction) (**Table 3**). The curve fitting diagram of stratified analysis showed that diabetes had a more significant effect on cervical cancer among those younger than 50 years old (**Figure 3A**) and other high-risk HPV infection types other than HPV16 and 18 (**Figure 3B**). We generated an E-value to assess the sensitivity to unmeasured confounding. The primary findings were robust, unless an unmeasured confounder existed with an OR greater than 6.03.

**Table 3:** Subgroup analysis of the association between HbA1c on cervical carcinoma.

Sub-group	n	CC (%)	OR (95% CI)	*p*-value	*p* for interaction
Age					0.0748
Age<50					
HbA1c <5.7	450	13 (2.89%)	Reference		
5.7≤HbA1c<6.5	95	10 (10.53%)	3.95 (1.68, 9.31)	0.0017	
HbA1c≥6.5	6	1 (16.67%)	6.72 (0.73, 61.70)	0.0920	
Age≥50					
HbA1c <5.7	114	11 (9.65%)	Reference		
5.7≤HbA1c<6.5	101	9 (8.91%)	0.92 (0.36, 2.31)	0.8525	
HbA1c≥6.5	25	5 (20.00%)	2.34 (0.73, 7.47)	0.1509	
HPV type					0.8964
Other high-risk types					
HbA1c <5.7	276	8 (2.90%)	Reference		
5.7≤HbA1c<6.5	99	6 (6.06%)	2.16 (0.73, 6.39)	0.1636	
HbA1c≥6.5	18	3 (16.67%)	6.70 (1.61, 27.86)	0.0089	
16/18					
HbA1c <5.7	288	16 (5.56%)	Reference		
5.7≤HbA1c<6.5	97	13 (13.40%)	2.63 (1.22, 5.69)	0.0140	
HbA1c≥6.5	13	3 (23.08%)	5.10 (1.28, 20.38)	0.0212	

## Discussion

Our findings highlight the clinical utility of screening for hyperglycemia in patients at high-risk for cervical cancer, and HbA1c screening should be considered as part of the clinical management of patients at high-risk for cervical cancer. Our study found that women with diabetes who had HGSIL and high-risk HPV infection are associated with cervical cancer after adjusting for age, adverse maternal history, vaginitis, and type of HPV infection. Subgroup analyses showed consistent associations between diabetes and cervical cancer in women with different characteristics. Our study shows that high blood glucose level may be a catalyst for the occurrence of cervical cancer in HGSIL patients with high-risk HPV infection, and provides clinical epidemiological evidence for in-depth understanding of the pathogenesis of cervical cancer.

Some studies suggest that diabetes is closely related to cervical cancer, especially genetic susceptibility to type 2 diabetes is associated with a high incidence of cervical cancer (8–13); other studies have found no statistically significant association between diabetes and cervical cancer (14–18). A retrospective cohort study of 328,994 diabetic and 327,572 nondiabetic participants found that newly diagnosed cases of type 2 diabetes mellitus (T2DM) (within 3 months) had a significantly increased risk of cervical cancer (HR=3.46, 95% CI:1.10-10.86, p=0.03). However, patients with T2DM were not at higher risk after the first 3 months compared with patients without T2DM (HR=1.10 95% CI: 0.79-1.53, p=0.57) (18).

Different studies have shown that the risk of cervical cancer in women with diabetes has increased to varying degrees. A cohort study of all women (n=2,508,321) born in Denmark between 1916 and 2001 pointed out that the incidence of cervical cancer in women with diabetes increased (IRR=1.13, 95% CI: 1.00-1.28) (20). In a meta-analysis of 19 studies, the relative risk of cervical cancer in women with diabetes was 1.34 (95% CI, 1.10-1.63) (21). In another study including 397,783 adults, after adjusting for age, body mass index, ethnicity, lifestyle, and physical activity, the prevalence of cervical cancer in the diabetic group was 30% higher than in the non-diabetic group (*p*=0.0011) (22). A study reported that in patients with type 2 diabetes, a 1% increase in HbA1C was associated with a 1.18-fold increased risk of cancer (HR=1.18, 95% CI: 1.04–1.33, *p* = 0.0102) (23).

Our research is based on a cross-sectional study of cervical clinics in China’s largest academic women’s hospital. We found that diabetes was associated with cervical cancer in high-risk groups of cervical cancer. Our findings explain why no relationship between diabetes and cervical cancer has been found in previous studies. Diabetes can promote cancer development on top of HPV infection. Our study suggests that monitoring of blood glucose control should be strengthened in high-risk groups, and histological diagnosis should be performed as soon as possible in diabetic patients with high-grade lesions and high-risk HPV infection to control hyperglycemia.

The mechanisms by which diabetes associated with cervical cancer may involve complex interactions, and some features of diabetes may explain this oncogenic predisposition, including hyperglycemia, hyperinsulinemia, chronic inflammatory states, and compromised immune systems (24). Hyperglycemia is associated with increased susceptibility to viral infection and cell-mediated immunodeficiency, which may impair the clearance of HPV infection, thereby promoting the progression of precancerous lesions and cancer (20). Hyperglycemia may promote tumor angiogenesis by upregulating microRNA-467, an inhibitor of the anti-angiogenic protein thrombospondin-1 (8). Hyperglycemia accelerates the process of glycolysis and promotes cancer cell proliferation through the increase of glucose transporter-1 in cancer cells. High levels of glucose promote cancer cell invasion and metastasis by stimulating epithelial-mesenchymal transition (EMT) (25). Enhanced expression of vascular endothelial growth factor can be induced under hyperglycemia, which is associated with vascular invasion, metastasis and tumor invasiveness (26). Hyperglycemia can activate various signaling pathways and gene expression associated with cancer cell proliferation, invasion and migration (27). In addition, poor glycemic control can lead to the production of advanced glycation end products, leading to oxidative stress and DNA damage (26). Poor glycemic control can lead to hyperinsulinemia, and insulin may exert mitogenic effects through the insulin-like growth factor 1 (IGF-1) receptor, thereby stimulating cell proliferation and inhibiting apoptosis (7, 8, 26). DM patients with poor glycemic control are often characterized by an enhanced chronic inflammatory state with increased levels of inflammatory cytokines TNF-α and IL-6. The microenvironment disrupted by chronic inflammation is critical for the progression of HPV-infected cells to cancer, and the coexistence of diabetes and HPV infection may create an inflammatory microenvironment to promote cervical cancer cell growth (8, 26, 28). Diabetes is not only a metabolic disease but also has profound and persistent effects on immune cell function (29). The proliferation of T cells and macrophages in patients with diabetes is altered, and the functions of NK cells and B cells are impaired, manifesting as innate and adaptive immune abnormalities (30), and the impaired immune function may be involved in the carcinogenesis of cervical cells. Combined with the above mechanistic studies, our study further provides clinical evidence that diabetes is a potential cause of cervical cancer.

Our study is a large cross-sectional study using relatively recent data (2019–2022) reflecting current disease patterns and trends; adjustments were made for risk factors including age, history of adverse, vaginitis, and HPV infection to reduce selection bias. Our data provide additional evidence for clinicians that women with diabetes who are infected with HGSIL and high-risk HPV types are associated with cervical cancer. The use of single and stable glycated hemoglobin to assess glycemic status is more clinically attractive. Our subjects were from the largest women’s teaching hospital in China, and all patients were reviewed pathologically by the same group of pathologists, ensuring stability and consistency in the interpretation of Pap smear and pathology sections.

Our study also has some limitations. First, cross-sectional data cannot be used to determine causality. Second, retrospective studies are imperfect and lack information on lifestyle-related factors such as high-risk sexual behavior and smoking. However, the smoking rate among Chinese women is about 2.1%, which is at a very low level (31). These unmeasured confounds may lead us to overestimate the association between diabetes and cervical cancer. However, we used E-value sensitivity analysis to quantify the potential impact of unmeasured confounders (32) and found that unmeasured confounders were unlikely to explain the overall association (E-value=6.03). Next, our study is a single-center study of high-risk patients with cervical cancer in China, and more multi-center studies are needed to confirm in the future. Thirdly, for diabetes, we did not differentiate between type 1 and type 2 diabetes, and we used only HbA1c for classification of prediabetes or diabetes. Finally, although infection from HPV has been the leading cause of cervical cancer, HPV-negative cervical cancer accounts for approximately 3 - 8% of all cases (33). The annual screening report in Belgium suggests that 15% of the cervical cancers were HPV negative. Frequent reasons for false negative HPV tumors are the used HPV testing methods and the misclassification of endometrial cancers or metastasis as cervical cancers. Other explanations are the loss of HPV expression (34). Of the 918 women screened for HGSIL in our study, 113 tested negative for HPV. In the future, we will pay more attention to the relationship between diabetes and HPV-negative cervical cancer.

## Conclusion

The results of this explorative study have important clinical and public health implications, more prospective cohort studies are needed in the future to verify our hypothesis. Our study found that women with diabetes who had HGSIL and high-risk HPV infection are associated with cervical cancer. This discrepancy suggests that clinicians need to pay attention to glycemic control in this high-risk group. Our results highlight the clinical utility of screening for hyperglycemia in patients at high-risk for cervical cancer, and we should consider HbA1c screening as part of the clinical management of patients at high risk for cervical cancer and recommend close monitoring of this population monitor during routine follow-up. We recommend increasing the cervical cancer index of suspicion in patients with diabetes and enhancing cancer screening recommendations for early detection of cervical cancer in this group of patients.

## Data availability statement

The original contributions presented in the study are included in the article/supplementary material. Further inquiries can be directed to the corresponding author.

## Ethics statement

The studies involving human participants were reviewed and approved by the ethics committee of Obstetrics and Gynecology Hospital of Fudan University (2022-78). Written informed consent for participation was not required for this study in accordance with the national legislation and the institutional requirements.

## Author contributions

CYY analyzed the data, drafted the manuscript and contributed to study design. CYZ contributed to data collation. CMY and HJ revised the article. All authors reviewed the manuscript, edited it for intellectual content, and gave final approval for this version to be published.

## Funding

This work was supported by the program for National Natural Science Foundation of China (No. 81902131) and Shanghai “Rising Stars of Medical Talents” Youth Development Program (SHWRS (2020)_087).

## Conflict of interest

The authors declare that the research was conducted in the absence of any commercial or financial relationships that could be construed as a potential conflict of interest.

## Publisher’s note

All claims expressed in this article are solely those of the authors and do not necessarily represent those of their affiliated organizations, or those of the publisher, the editors and the reviewers. Any product that may be evaluated in this article, or claim that may be made by its manufacturer, is not guaranteed or endorsed by the publisher.
